# Minimally invasive simple prostatectomy in the era of laser enucleation for high-volume prostates: A systematic review and meta-analysis

**DOI:** 10.1080/2090598X.2020.1789809

**Published:** 2020-08-11

**Authors:** Josselin Abi Chebel, Julien Sarkis, Elie El Helou, Elie Hanna, Georges Abi Tayeh, Albert Semaan

**Affiliations:** Department of Urology, Hôtel-Dieu de France Hospital, Beirut, Lebanon

**Keywords:** Laser, enucleation, BPH, adenomectomy, simple prostatectomy

## Abstract

**Objective:**

To perform a systematic review and meta-analysis of available prospective and retrospective studies comparing the minimally invasive (laparoscopic or robot-assisted) simple prostatectomy (MISP) and laser enucleation of the prostate for treating male lower urinary tract symptoms in high-volume prostates, as laser enucleation of the prostate is the new trend for treating high-volume prostates (>80 mL) but many urologists now prefer MISP.

**Methods:**

A systematic search was done using the Medical Literature Analysis and Retrieval System Online (MEDLINE) and Cochrane databases in June 2019, with research terms including: ‘laser’, ‘laparoscopy’, ‘enucleation’, ‘BPH’, ‘simple prostatectomy’, ‘Millins’, and ‘adenomectomy’. The meta-analysis was conducted according to the Preferred Reporting Items for Systematic Reviews and Meta-Analyses (PRISMA) guidelines.

**Results:**

Of 38 screened articles, six were analysed and a total of 975 men were included. The average operative time, length of stay and catheterisation time were significantly shorter in the laser enucleation group (*P* = 0.006, *P* < 0.001 and P < 0.001, respectively). The amount of prostatic tissue removed during surgery was comparable between both the laser enucleation and MISP groups (*P* = 0.39). The International Prostate Symptom Score, prostate-specific antigen level, maximum urinary flow rate and post-void residual urine volume were also comparable at 3 months. Finally, similar transfusion rates and Clavien–Dindo complication rates were observed (*P* = 0.08 and *P* = 0.41, respectively).

**Conclusion:**

This systematic review of the literature and meta-analysis provide a further demonstration of the safety and effectiveness of both laser enucleation and MISP. While laser enucleation had a shorter catheterisation time and hospital stay than MISP, the latter still had unique and specific indications.

**Abbreviations:** ELEP: eraser laser enucleation of the prostate; HoLEP: holmium laser enucleation of the prostate; PRISMA: Preferred Reporting Items for Systematic Reviews and Meta-Analyses; PVR: post-void residual urine volume; Q_max_: maximum urinary flow rate; (L)(MI)(RA)SP: (laparoscopic) (minimally-invasive) (robot-assisted) simple prostatectomy

## Introduction

BPH affects 70% of men aged between 60 and 69 years, and has a significant impact on a patient’s quality of life, particularly for those with moderate-to-severe LUTS with prostate volumes of >80 mL [1]. While the first-line treatment of BPH is usually medical therapy, surgical intervention is needed in refractory conditions, as well as in complicated cases (recurrent UTIs, bladder calculi, bladder diverticulum, refractory haematuria, and dilation of the upper tract) [2].

Laser endoscopic enucleation (mainly holmium laser enucleation of the prostate [HoLEP] [3], eraser laser enucleation of the prostate [ELEP] [4] and thulium vapo-enucleation of the prostate [ThuVEP] [5]) of the prostate is becoming the new trend for BPH treatment for prostates of >80 mL, with comparable results and a better safety profile than the classical open simple prostatectomy (SP) [6–9]. Nonetheless, a considerable number of urologists are relying on SP as the treatment of choice for high-volume BPH, especially with the development of minimally invasive laparoscopic and robot-assisted SP (LSP and RASP) that benefit from the same perioperative results of the open technique, but with a better safety profile [10,11].

The aim of the present study was therefore to systematically review and analyse the evidence and compare the effectiveness and safety between LSP/RASP and laser enucleation for treating male LUTS due to high-volume prostates.

## Methods

To our knowledge, there is no systematic review of the literature comparing minimally invasive SP (MISP) with laser enucleation for the treatment of BPH. A systematic search was conducted according to the Preferred Reporting Items for Systematic Reviews and Meta-Analyses (PRISMA) checklist [12]. The search was done using the Medical Literature Analysis and Retrieval System Online (MEDLINE) and Cochrane library databases on the 23 June 2019. Specific search terms included, but were not limited to: ‘laser’, ‘laparoscopy’, ‘enucleation’, ‘BPH’, ‘simple prostatectomy’, ‘Millins’, and ‘adenomectomy’. Phrases were combined using Boolean operators (AND, OR) to augment the search.

### Data extraction and analysis

All prospective or retrospective studies comparing laser prostate enucleation with either LSP or RASP were included in this review. We excluded non-comparative studies, reviews and comments, as well as studies that included prostates of <80 mL. The list of the potential studies was then reviewed by the authors and data were collected. The identification of relevant studies, the selection based on the criteria just described, and the subsequent data extraction were performed independently by two of the authors; conflicts were resolved by a third author. The meta-analytic software RevMan 5.3 (Cochrane, London, UK) was employed. Both random- and fixed-effects analyses were performed. The random-effects analyses were considered the lead approach, as they make fewer assumptions.

### Outcome measures

The primary endpoints analysed were postoperative outcomes: the maximum urinary flow rate (Q_max_), postoperative PSA level, post-void residual urine volume (PVR) and the IPSS. Postoperative dysuria and urinary incontinence were also reviewed. Secondary outcomes included perioperative parameters such as operative time, catheterisation time, length of stay, amount of tissue removed, blood loss, and complications according to the Clavien–Dindo classification system [13].

## Results

**Figure 1.**
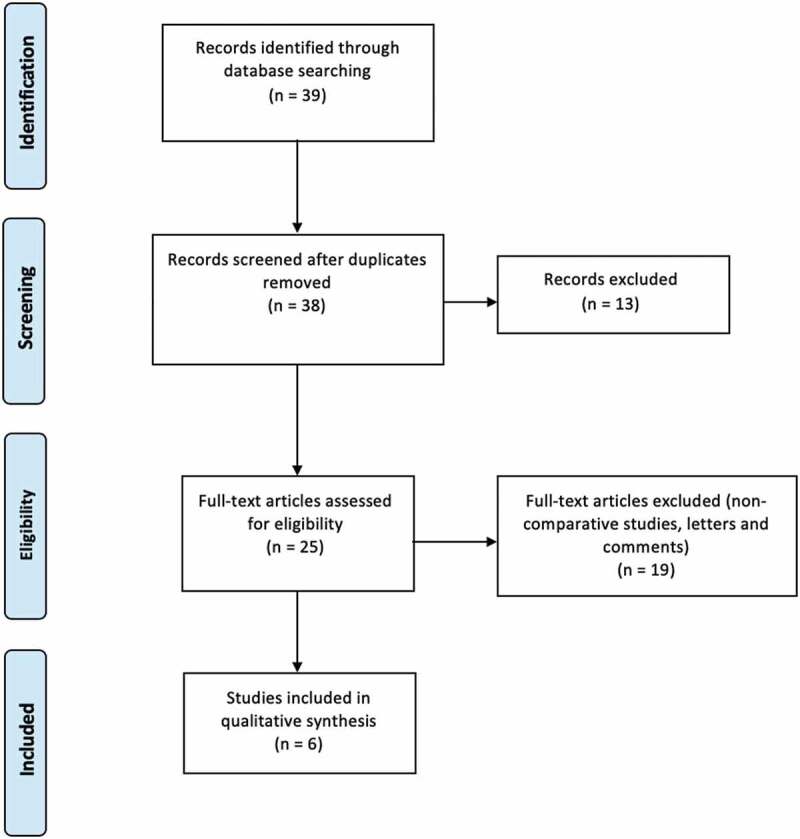
Study PRISMA flow chart

In total, 38 articles were screened, from which six met our predefined inclusion criteria. Those six articles were analysed: five were retrospective trials and only one was a prospective trial. One study [14] was reviewed but was not included in the meta-analysis due to an absence of dispersion measures. The study’s PRISMA flow chart is presented in Figure 1.

### Characteristics of included studies

All studies were conducted either in Europe or the USA, and published between 2015 and 2018. They included a total of 975 patients, of which 759 underwent laser enucleation (704 HoLEP, 20 ELEP, and 35 ThuVEP); 216 underwent MISP (68 LSP and 148 RASP). The patient demographics of both groups were comparable. All patients had a prostate volume measurement performed by a radiologist via TRUS. The surgical techniques used in the studies were either transcapsular (Millin) prostatectomy or transvesical (Freyer) prostatectomy. For HoLEP, the conventional Gilling technique was used. The summary of the preoperative parameters for the different studies are listed in Table 1 [1,14–18].

**Table 1.** Summary of preoperative parameters

		Patients, *n*	Prostate volume, mL, mean (SD)	IPSS, mean (SD)	Q_max_, mL/s, mean (SD)	PVR, mL, mean (SD)	PSA level, ng/ml, mean (SD)
Baldini et al. [16]	HoLEP	39	83.9 (179.9)	21.1 (6.37)	8.2 (5.2)	137.1 (18.5)	7.2 (4.6)
	LSP	28	120.5 (197.1)	19.8 (13.5)	7.5 (4.7)	159.4 (47.1)	8.4 (7.7)
	*P*		<0.001	0.607	0.702	0.675	0.430
Juaneda et al. [14]	HoLEP	20	126.5	21	6.5	–	–
	LSP	20	127.5	23.5	8.9	–	–
	*P*		0.91	–	*–*	*–*	*–*
Lusuardi et al. [15]	ELEP	20	96.1 (35.9)	28.4 (4.95)	6.7 (2.6)	173.7 (82.5)	8.07 (3.7)
	LSP	20	94.0 (22.4)	27.7 (4.96)	7.8 (2.27)	142.5 (69.6)	7.5 (3.3)
	*P*		0.83	0.64	0.18	0.2	0.61
Nestler et al.* [18]	ThuVEP	35	95.2 (37.1)	20.4 (3.9)	–	–	–
	RASP	35	104.8 (41.8)	22.6 (3.9)	–	–	–
	*P*		0.41	0.5	–	*–*	*–*
Umari et al.* [17]	HoLEP	45	131.1 (28.3)	19.9 (6.9)	8.6 (5.3)	107.4 (98.8)	9.7 (8.6)
	RASP	81	144.4 (59.6)	24.2 (6.0)	8 (4.5)	75.8 (43.7)	8.1 (6.3)
	*P*		0.06	0.05	0.5	0.2	0.6
Zhang et al. [1]	HoLEP	600	–	20 (7)	–	–	–
	RASP	32	–	24 (4)	–	–	–
	*P*		*–*	0.21	*–*	*–*	*–*

*Median values presented in Nestler et al. and Umari et al. were converted to mean ± SD (Luo D, Wan X, Liu J, et al. Optimally estimating the sample mean from the sample size, median, mid-range, and/or mid-quartile range. Stat Methods Med Res. 2018;27:1785–805).

### Perioperative endpoints

**Figure 2.**
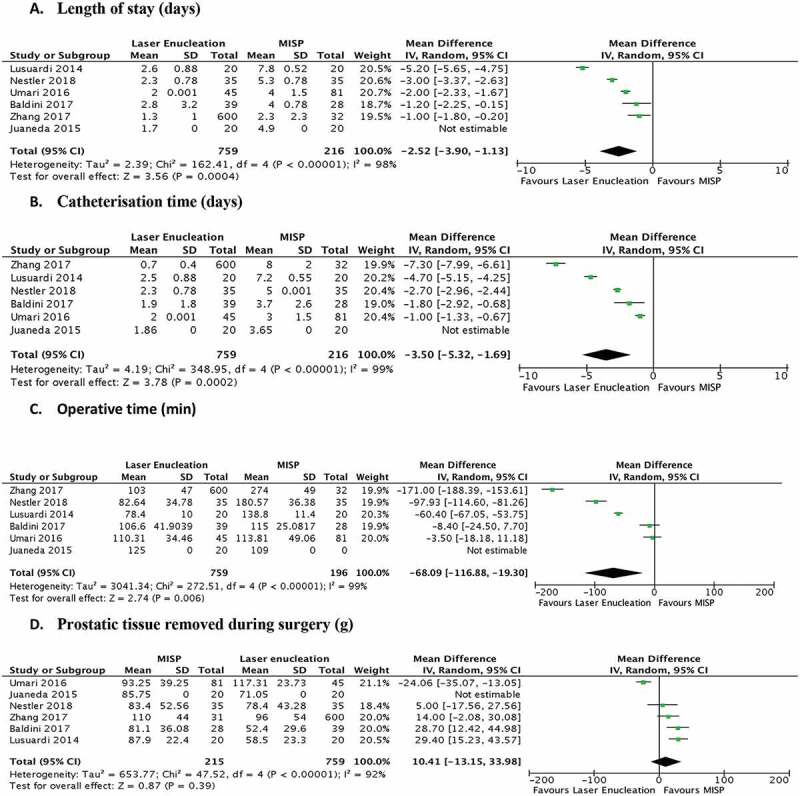
Forrest plots comparing perioperative parameters. Average length of stay, catheterisation time and operative time were all in favour of laser enucleation, while volume of prostatic tissue resected was comparable between both groups

The perioperative outcomes were available from all six studies (Figure 2). The average operative time, with a mean difference of 68.09 min (*P* = 0.006, Figure 2(c)), length of stay (mean difference of 2.52 days, *P* < 0.001, Figure 2(a)) and catheterisation time (mean difference of 3.5 days, *P* < 0.001, Figure 2(b)), was significantly shorter in the laser enucleation group when compared to MISP. The amount of prostatic tissue removed during surgery, even though higher in the MISP group, was statistically comparable [*P* = 0.39, mean difference of 10.41 g (95% CI – 13.15, 33.98); Figure 2(d)].

### Postoperative endpoints

**Figure 3.**
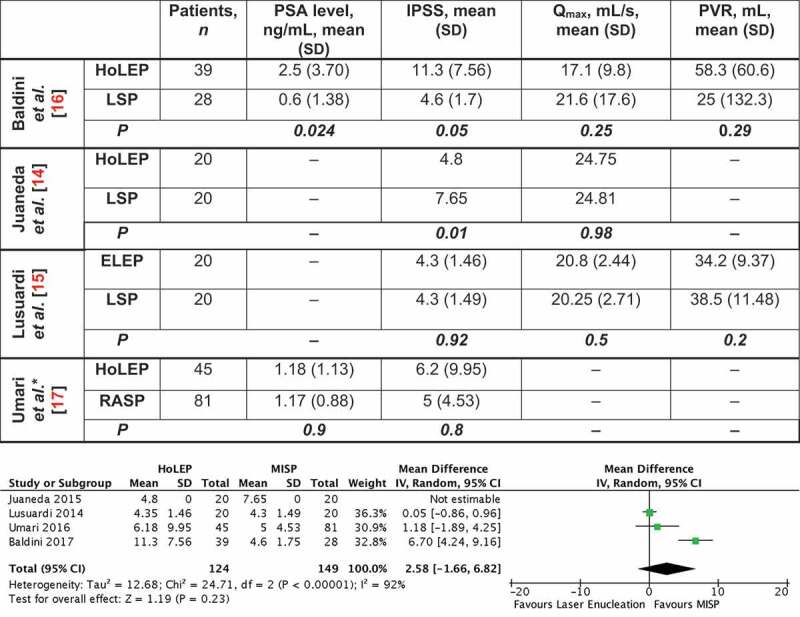
Follow-up at 3 months. Forrest plot comparing IPSS at 3 months after surgery indicates no statistical difference between both groups. Meta-analysis of remaining outcomes showed comparable postoperative Q_max_ [mean difference – 0.7 mL/s (95% CI – 4.98, 3.57)], PSA level [mean difference 0.17 ng/mL (95% CI – 0.20, 0.53)] and PVR [mean difference – 3.73 mL (95% CI – 10.18, 2.71)]

A 3-month follow-up was completed in four studies, with statistically comparable post-surgical outcomes (IPSS, PSA level, Q_max_ and PVR) on meta-analysis (Figure 3 [14–17]). Only one study [15] continued follow-up to 6 months after surgery, with comparable IPSS [mean (SD) 4.05 (1.23) in the ELEP group vs 4.70 (0.73) in the LSP group, *P* = 0.052], but a statistically lower PVR in favour of laser enucleation [mean (SD) 33.9 (9.26) vs 44.5 (15) mL, *P* = 0.011). On the other hand, Baldini et al. [16] reported that eight of 39 (25%) patients who underwent HoLEP reported postoperative pollakiuria and urinary urgency, compared to two of 28 (7.1%) in the LSP group. Umari et al. [17] also reported that four of 45 (8.9%) patients reported transient urinary incontinence following HoLEP, while only one of 81 (1.2%) complained of transient incontinence following RASP. Only one study [15] evaluated quality of life at 3 and 6 months, showing comparable results (*P* = 0.84).

*Median values presented in Umari et al. were converted to mean ± SD (Luo D, Wan X, Liu J, Tong T. Optimally estimating the sample mean from the sample size, median, mid-range, and/or mid-quartile range. Stat Methods Med Res. 2018;27:1785–1805).

### Complications and blood loss

**Figure 4.**
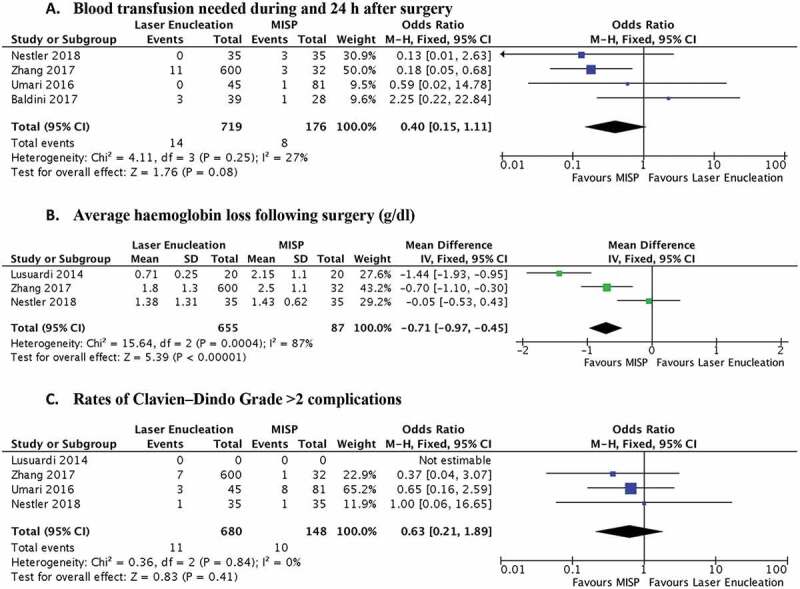
Forrest plots comparing surgical complications. Blood loss was statistically higher in the MISP group, but did not translate to a higher transfusion rate. Both surgeries had comparable Clavien–Dindo Grade >2 complications

There was statistically significantly higher blood loss in the MISP group (*P* < 0.001, Figure 4(b)), but without a significantly higher rate of blood transfusions (*P* = 0.08, Figure 4(a)). While Nestler et al. [18] reported a lower complication rate in the ThuVEP group when compared to MISP (one of 35 vs nine of 35), analysis of Clavien–Dindo complications of Grade ≥3 showed comparable results between the laser enucleation and MISP groups (*P* = 0.41, Figure 4(c)).

## Discussion

With recent technical developments in laser technology, endoscopic enucleation of the prostate and SP have both become the ‘gold standard’ for surgical therapy of high-volume BPH [19]. At the same time, LSP and RASP (MISP) are overcoming the more classical open approach, mainly due to their better safety profile. However, even in the era of minimally invasive surgery, few studies exist comparing MISP with endoscopic enucleation of the prostate. We therefore performed the present systematic review of the literature with subsequent meta-analysis to better judge these two procedures. We found that MISP (either LSP or RASP) and enucleation of the prostate are equally efficient in terms of postoperative Q_max_, PVR and IPSS, therefore showing similar impact on relieving the BOO. This is added to the fact that both techniques resected statistically comparable amounts of prostatic tissue. Additionally, both surgeries appear to have few postoperative complications (11/680 Clavien–Dindo Grade >2 complications in the laser enucleation group and 10/148 in the MISP group, *P* = 0.41), making them safe procedures when performed by expert surgeons. And although haemoglobin loss was statistically higher in the MISP group, it did not translate to a higher transfusion rate (probably explained by the pneumoperitoneum effect in preventing major venous bleeding during laparoscopic or robotic interventions). It should be noted, however, that two studies reported higher postoperative dysuria and incontinence rates in the laser enucleation group.

The main differences found between the two techniques concerned the perioperative outcomes: patients undergoing laser enucleation of the prostate had a shorter operative time and Foley catheterisation time, and were consequently discharged sooner from hospital.

Despite the advantages of laser enucleation, MISP is preferable for specific indications: a very interesting indication for LSP or RASP is the concomitant treatment of bladder diverticulum or bladder stone [20]. These cases are rarely treated via a transurethral approach, as large bladder stones would add a significant operative time if treated with laser techniques.

In addition, while both techniques have learning difficulties in common [16], RASP could be considered an interesting option for surgeons who have completed the learning curve by performing an adequate number of robot-assisted radical prostatectomies. In comparison, surgeries that involve laser enucleation of the prostate, e.g. the HoLEP technique, have a long learning curve with variability in performance, even for a surgeon with extensive experience [21,22].

A further issue that needs assessment is the financial aspect of both procedures. For instance, Juaneda et al. [14] evaluated the separate cost of HoLEP and LSP, with a mean cost of ‎€2871 (Euros) for HoLEP and ‎€4706 for LSP. This difference is mainly due to the fact that the hospital stay is much shorter when using the HoLEP technique. In a hospital where laparoscopy is available and in low-volume centres that cannot afford the expenses of a new holmium generator, LSP may be more affordable than HoLEP. More studies assessing real data and cost analysis (capital costs of equipment, hospital stay and care costs, complications and operative time) are needed.

### Strengths and limitations

This is the first literature review comparing LSP or RASP with laser enucleation, mainly HoLEP; the review is strengthened by its systematic approach. Another positive attribute is the large number of patients included (975). The main limitation is the small number of studies included, and the retrospective design of most of them. Furthermore, sexual function is an important patient-related outcome that was not assessed in the analysed studies. The variability of laser enucleation arms across the studies (LSP, RASP, HoLEP, ELEP, ThuVEP) made the comparison uneven. Further prospective randomised studies are needed to fully identify any significant difference among all of the techniques.

## Conclusion

For men requiring a surgical treatment for their BPH with large-volume prostates of >80 mL, MISP and laser enucleation both appear to be effective and safe procedures. While laser enucleation showed a better perioperative profile, such as shorter catheterisation time and hospital stay compared with that of MISP, the latter has specific indications when bladder diverticulum or stones are present. These results can help physicians choose the best surgical approach for patients with high-volume BPH.

Finally, more prospective randomised studies, as well as meta-analyses comparing MISP and laser enucleation, are necessary to define the standard surgical treatment for large prostates.
